# A Global and Local SHAP-Driven Interpretable Framework for Early Hospital Admission Prediction With Machine Learning at Emergency Department Triage

**DOI:** 10.7759/cureus.111163

**Published:** 2026-06-19

**Authors:** Adam E Brown, Chance W Marostica, Nicole R Hodgson, Wayne A Martini

**Affiliations:** 1 Emergency Medicine, Mayo Clinic Alix School of Medicine, Phoenix, USA; 2 Biomedical Sciences, Mayo Clinic Graduate School of Biomedical Sciences, Phoenix, USA; 3 Emergency Medicine, Mayo Clinic Arizona, Phoenix, USA

**Keywords:** catboost, emergency department, explainable artificial intelligence (xai), hospital admission, interpretable machine learning, shap, triage, xgboost

## Abstract

Background: Emergency department (ED) crowding and constrained hospital resources drive the need for early risk stratification to efficiently identify patients requiring inpatient admission or escalation of care. Machine learning (ML) models are promising as helpful tools for admission prediction, yet there remains limited literature exploring the integration of explainability techniques to enhance the transparency of these models for clinicians and resource allocation staff end-users. Explainability, in this context, refers to techniques that show which patient features contributed most to each individual prediction and to the model's overall behavior.

Objectives: To develop ML models predicting hospital admission at ED triage and to demonstrate population-level and individual-level explainability as a step toward building trust and fair evaluation of AI-assisted triage models.

Methods: We conducted a retrospective study of ED visits (2020-2024) across three tertiary-care EDs. We extracted structured triage data, including patient demographics, vital signs, Emergency Severity Index (ESI), arrival mode, and comorbidities from the electronic medical record. Six models were developed and evaluated on a temporally distinct holdout test set. SHapley Additive exPlanations (SHAP) were applied to the two top-performing models (CatBoost and XGBoost) on a population and individual prediction level.

Results: The training cohort included 620,729 visits (32.4% admitted); the test cohort included 183,789 visits (32.3% admitted). CatBoost achieved an area under the receiver operating characteristic curve (AUC) of 0.870 (F1 0.701; sensitivity 79.0%; specificity 78.0%). XGBoost achieved an AUC of 0.848. ESI level, vital signs, means of arrival, and presenting complaints were among the most influential predictors. Post-hoc Platt scaling reduced the Brier score from 0.146 to 0.134 and corrected a systematic over-prediction of admission probability (calibration intercept moved from -0.69 to 0.02).

Conclusions: ML models using triage data predict hospital admission with good discrimination. Population-level and individual-level SHAP provide transparency that may facilitate clinician trust and fair model evaluation.

## Introduction

Emergency departments (EDs) are increasingly challenged by crowding and resource constraints, making early identification of patients who will require hospital admission a clinical and operational priority [1-5]. Patients who ultimately require inpatient care benefit from expedited evaluation and disposition, as delays in admission from the ED can worsen outcomes [2,6]. Traditional triage systems, such as the Emergency Severity Index (ESI), assign an acuity level from 1 (highest) to 5 (lowest) based on initial presentation [7]. While ESI effectively stratifies patients by illness severity, it is a coarse tool and may not reliably identify patients who will require admission [8,9]. There remains a need for more nuanced, data-driven decision support tools at triage to predict hospital admission [10,11].

Prior studies have demonstrated the potential of using machine learning (ML) for ED triage decision support [12-15]. Predictive models have been developed to forecast clinically relevant outcomes such as which ED patients will require hospital admission or which patients may return for further evaluation after discharge [15-18]. Early research with logistic regression models using triage vital signs, demographics, or administrative data has demonstrated moderate performance (AUC 0.69-0.85) [1,13,19]. More advanced ML techniques, such as gradient boosting and neural networks, have further improved performance, showing that ML can learn non-linear relationships to make predictions aligning closer to clinical reasoning [18,20,21]. A study by Fernandes et al. (2020) applied ML, including natural language processing of chief complaints, to predict admissions to higher care units, reporting an AUC of 0.91 [15]. Similarly, studies by Fenn et al. (2021) and Pandey et al. (2025) found strong model discrimination ability for predicting admissions and need for escalation of care [18,20].

While ML clinical decision tools at ED triage have shown promising results, there has been slow adoption and engagement with such models due to a lack of trust and difficulty with assessing the added value of such prediction models [22,23]. There also currently exists limited research that combines rigorous model evaluation with eXplainable Artificial Intelligence (XAI) techniques applied at both the population and individual prediction levels [24-26]. This need for explainability is critical for building clinician trust, evaluating whether a model's reasoning aligns with clinical logic, and has been shown to correlate with end-user engagement [22,23]. Explainability methods are also increasingly emphasized in updated FDA quality management reporting guidelines for ML models in the healthcare setting [27].

The primary objective of this study was to develop and evaluate ML models that predict hospital admission from data available at the moment of ED triage. The secondary objectives were to compare model performance across six classifier families to identify the strongest discriminative approach; to demonstrate population-level and individual-level explainability of the top-performing models using SHapley Additive exPlanations (SHAP); and to discuss operational and trust-related implications that follow from coupling explainability with predictive performance. Clinician trust and operational improvement were framed as motivating considerations rather than directly tested endpoints.

## Materials and methods

Study design and setting

We performed a retrospective cohort study of ED visits from January 1, 2020, through December 31, 2024, at three academic tertiary-care EDs of Mayo Clinic Hospital sharing a common electronic health record (EHR) with campuses in Arizona, Florida, and Minnesota. The study included all patients evaluated in the ED who were subsequently either admitted to the hospital (including observation status) or discharged. We excluded encounters with dispositions such as "left without being seen," "transfer to an outside facility," or "dead on arrival" to focus specifically on the binary classification of admission versus discharge. This project was reviewed by the institutional IRB and deemed exempt research with a waiver of informed consent (IRB #25-002035).

Cohort construction and data extraction

All data were obtained from the EHR data warehouse via structured query language (SQL) queries. We queried the ED encounters database for all visits during the study period at the three study sites. For each encounter, we retrieved the unique encounter ID, patient ID, timestamps (arrival and ED departure), and triage acuity level (ESI score). Each encounter was labeled as "admitted" if the patient was admitted to the hospital for inpatient care or placed in observation status, and "discharged" if the patient was discharged from the ED. Using SQL joins, we gathered structured clinical data available at triage for each encounter. This included patient demographics (age, sex), mode of arrival, vital signs at triage (heart rate, systolic blood pressure, diastolic blood pressure, respiratory rate, oxygen saturation, body temperature), complaint category, and the ESI triage level. We also extracted each patient's active medical comorbidities from the EHR problem list and prior diagnoses at the time of the specific ED visit. Key comorbid conditions were flagged as binary features: heart failure, myocardial infarction or coronary artery disease, diabetes, chronic kidney disease, chronic lung disease (chronic obstructive pulmonary disease (COPD) or asthma), active cancer or malignancy, history of stroke or transient ischemic attack, history of pulmonary embolism, and organ transplant history. Data processing was performed in Microsoft SQL Server (Microsoft Corporation, Redmond, WA, USA) and Python (Python Software Foundation, Fredericksburg, VA, USA). A final analytic dataset was assembled with one record per ED visit, the binary outcome (admission vs. discharge), and 23 predictive features.

Missing data

Missing values for vital signs were minimal (<5% for any individual vital sign). For the XGBoost-imputed model, Random Forest, Logistic Regression, and Naive Bayes models, missing vital sign values were imputed using population medians. CatBoost and XGBoost (Raw) handle missing values natively, so imputation was not required for these models. Categorical features (ESI level, complaint category, reason for visit, means of arrival, ED site (Dept), and sex) were treated as pandas category dtypes and passed natively to CatBoost. For XGBoost and the other comparator models that do not handle categoricals natively, each categorical feature was label-encoded into integer codes, preserving the original level mapping, which was the same mapping used during training and at inference. No high-cardinality target encoding was performed.

Feature selection and model design

We included only structured features available at the time of triage, excluding laboratory results and radiology findings. In total, our models used 23 features: vital signs (6), ESI level, complaint category, means of arrival, reason for visit, ambulance arrival, ED site (Dept), age, sex, and existing comorbidity flags (9). Free-text chief complaint descriptions were not incorporated in the model development.

Complaint category and means of arrival are structured fields captured by the triage nurse at the time of ED registration. The reason for visit is a structured category assigned during the same triage workflow. These fields are determined before the admission decision and thus do not constitute direct outcome leakage; however, they may encode aspects of triage-nurse clinical judgment about acuity and may reflect site-specific or pattern-specific workflows. We retained these features as available-at-triage predictors and report them as such.

Model training, validation, and threshold optimization

The training cohort consisted of ED visits from January 1, 2020, through December 31, 2023, at all three ED sites. A temporally distinct holdout test set was constructed from all encounters between January 1, 2024, and December 31, 2024, ensuring that model performance was evaluated on completely novel data from a different calendar year.

Within the training data, 80% of encounters were allocated for model development and 20% for internal validation, stratified by outcome. Six models were developed: CatBoost, XGBoost-Raw (handling missing values natively), XGBoost-Impute (using imputed values for missing datapoints), Random Forest, Logistic Regression, and Naive Bayes. For CatBoost and XGBoost, hyperparameters (tree depth, learning rate, class weight scaling) were tuned empirically. Logistic Regression, Random Forest, and Naive Bayes were developed with minimal hyperparameter tuning.

Threshold optimization was performed on the training data by selecting the probability cutoff that maximized the F1 score, balancing sensitivity (recall) and positive predictive value (precision). This was done separately for each model. The resulting thresholds were approximately 0.50 for the admission prediction task. Final tuned models were then evaluated on the holdout test set using the training-derived threshold.

Analyses were conducted in Python 3.12 using pandas (2.x), scikit-learn (1.5.x), CatBoost (1.2.x), XGBoost (2.0.x), and SHAP (0.46.x). Final tuned hyperparameters for all six models are reported in Appendix 1, including random seeds. Patient-level data are restricted under institution data governance and are not publicly redistributable; aggregate metrics and the analytic methodology are sufficient to reproduce the reported analyses on a comparably structured triage dataset by external research groups.

Evaluation metrics

Model discrimination was assessed by the area under the receiver operating characteristic curve (AUC) and the area under the precision-recall curve (PR AUC). We report sensitivity, specificity, positive predictive value (PPV), negative predictive value (NPV), accuracy, and F1 score at the optimized threshold. Calibration was examined using calibration curves and the Brier score.

Post-hoc calibration

Post-hoc calibration of the champion CatBoost model was performed using Platt scaling (sigmoid) fitted on a held-out 20% stratified calibration split of the training cohort (random seed 42). Calibrated probabilities were generated for the temporally distinct 2024 holdout test set, and we report pre- and post-calibration Brier scores, log loss, calibration slope, and calibration intercept (Appendix 2). Isotonic regression was also examined as a non-parametric alternative for sensitivity. Calibration analyses were performed using scikit-learn's LogisticRegression for Platt scaling and IsotonicRegression for the isotonic method.

Explainability analysis

We applied SHAP to the CatBoost and XGBoost models to assess feature importance at the population level and to explain individual predictions. SHAP values provide a consistent, theoretically grounded method for attributing each feature's contribution to a given prediction. Population-level SHAP summary plots display the distribution of feature impacts across all predictions. Individual-level SHAP waterfall and force plots show how each feature contributed to a specific patient's predicted probability of admission. In practical terms, a SHAP value quantifies how much a given feature shifted the model's predicted probability of admission for that specific patient, relative to the model's average prediction.

## Results

Cohort characteristics

The training cohort included 620,729 ED visits across the three sites after exclusions and data cleaning. Of these, 201,286 (32.4%) resulted in hospital admission or observation, and 419,443 (67.6%) were discharged from the ED. The median patient age was 57 years (interquartile range (IQR) 34-72). Admitted patients were older (median 66 years, IQR 50-77) compared with discharged patients (median 51 years, IQR 29-68). Admitted patients were more likely to arrive by ambulance (30.2% vs. 11.0%) and to be triaged at higher acuity levels (ESI 1-2: 36.7% of admitted vs. 12.2% of discharged patients). Notably, 60.5% of admitted patients had an initial ESI of 3, indicating that standard triage acuity alone did not identify all patients requiring admission.

The test cohort (2024 data) included 183,789 ED visits; 59,422 (32.3%) resulted in admission. Demographic and clinical characteristics of both cohorts, stratified by disposition, are presented in Table 1.

**Table 1:** Baseline characteristics of the training and test cohorts, stratified by disposition. IQR: interquartile range; ESI: Emergency Severity Index; SBP: systolic blood pressure; DBP: diastolic blood pressure; SpO2, peripheral oxygen saturation; MI: myocardial infarction; CAD: coronary artery disease; CKD: chronic kidney disease; COPD: chronic obstructive pulmonary disease; TIA: transient ischemic attack; PE: pulmonary embolism

Characteristic	Training Admitted (n=201,286)	Training Discharged (n=419,443)	Test Admitted (n=59,422)	Test Discharged (n=124,367)
Age, median (IQR)	66 (50–77)	51 (29–68)	67 (50–77)	52 (30–70)
Female, n (%)	96,349 (47.9%)	229,183 (54.6%)	29,161 (49.1%)	68,704 (55.2%)
Ambulance arrival, n (%)	60,783 (30.2%)	46,189 (11.0%)	16,661 (28.0%)	12,997 (10.5%)
ESI 1, n (%)	5,220 (2.6%)	735 (0.2%)	1,512 (2.5%)	209 (0.2%)
ESI 2, n (%)	68,707 (34.1%)	50,456 (12.0%)	19,355 (32.6%)	13,757 (11.1%)
ESI 3, n (%)	121,833 (60.5%)	271,293 (64.7%)	37,197 (62.6%)	82,400 (66.3%)
ESI 4, n (%)	5,309 (2.6%)	94,796 (22.6%)	1,314 (2.2%)	27,436 (22.1%)
ESI 5, n (%)	17 (0.0%)	1,956 (0.5%)	13 (0.0%)	501 (0.4%)
SBP, mmHg, median (IQR)	126 (113–139)	130 (117–144)	125 (113–139)	129 (117–144)
DBP, mmHg, median (IQR)	72 (63–81)	78 (70–87)	73 (64–82)	78 (70–87)
Heart rate, bpm, median (IQR)	77 (68–89)	77 (67–89)	77 (67–88)	77 (67–88)
Respiratory rate, median (IQR)	17 (16–18)	18 (16–19)	16 (16–18)	18 (16–19)
SpO2, %, median (IQR)	97 (95–98)	98 (96–99)	97 (95–98)	97 (96–99)
Temperature, °F, median (IQR)	98.1 (97.7–98.3)	98.2 (97.9–98.5)	98.0 (97.7–98.2)	98.1 (97.7–98.4)
Heart failure, n (%)	34,977 (17.4%)	28,982 (6.9%)	9,021 (15.2%)	7,635 (6.1%)
MI or CAD, n (%)	36,061 (17.9%)	38,874 (9.3%)	9,748 (16.4%)	10,601 (8.5%)
Diabetes, n (%)	56,623 (28.1%)	68,924 (16.4%)	16,154 (27.2%)	20,393 (16.4%)
Chronic kidney disease, n (%)	32,953 (16.4%)	28,589 (6.8%)	8,458 (14.2%)	7,634 (6.1%)
COPD or asthma, n (%)	28,592 (14.2%)	69,207 (16.5%)	8,956 (15.1%)	20,167 (16.2%)
Cancer or malignancy, n (%)	68,214 (33.9%)	92,093 (22.0%)	20,985 (35.3%)	27,587 (22.2%)
TIA or stroke, n (%)	27,870 (13.8%)	30,818 (7.3%)	7,831 (13.2%)	8,637 (6.9%)
Pulmonary embolism, n (%)	13,318 (6.6%)	15,151 (3.6%)	4,127 (6.9%)	4,470 (3.6%)
Transplant history, n (%)	8,268 (4.1%)	6,307 (1.5%)	2,248 (3.8%)	1,733 (1.4%)

Model performance

**Figure 1:**
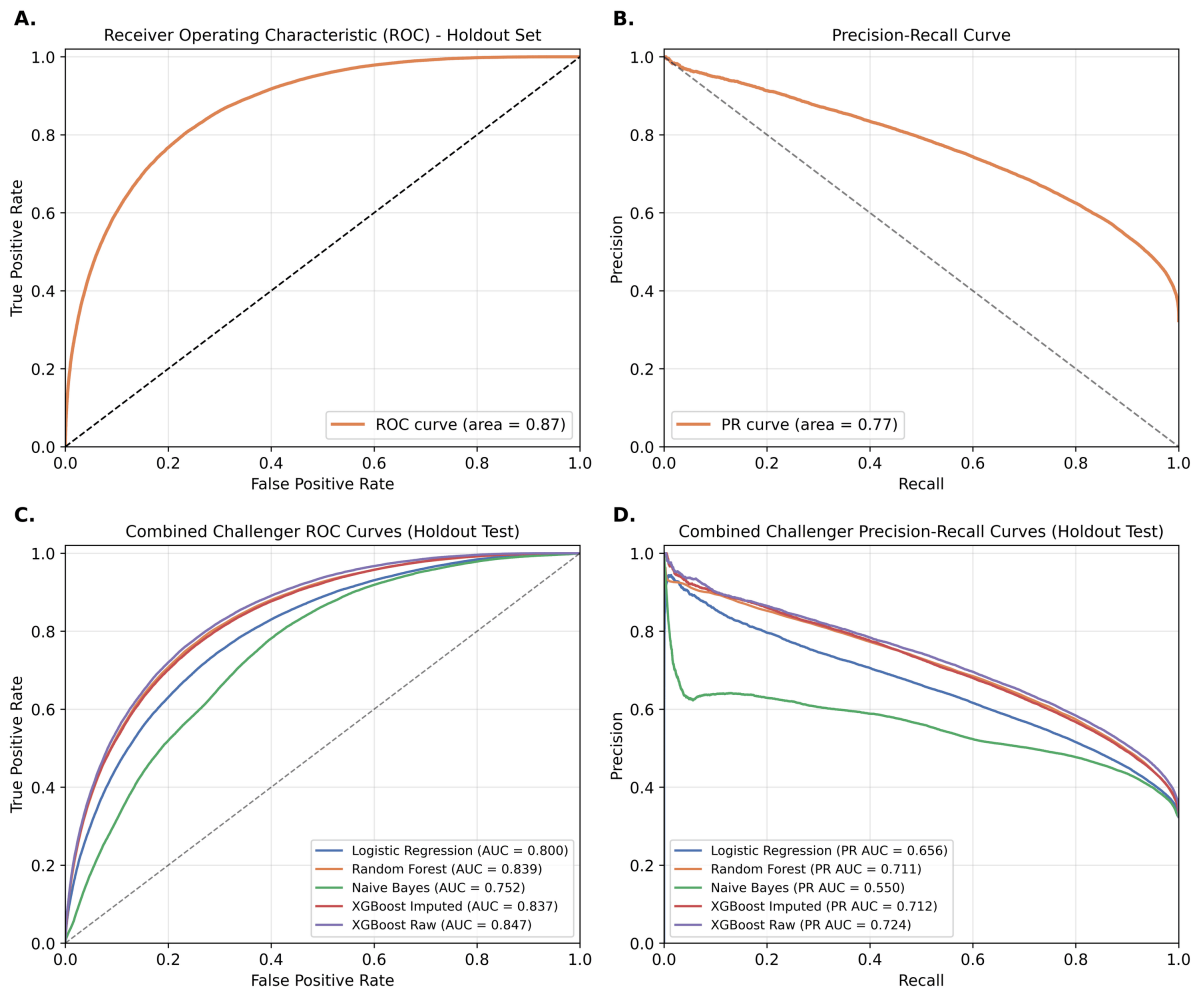
Receiver operating characteristic (ROC) curve and precision-recall (PR) curve for CatBoost and additional models on the holdout test set. A-B: Receiver operating characteristic (ROC) curve, precision-recall (PR) curve, for the champion CatBoost model on the holdout test set. The CatBoost model demonstrated strong discriminative performance with AUC = 0.870. C-D: ROC curves and PR curves for the five additional models (XGBoost Raw, XGBoost Imputed, Random Forest, Logistic Regression, Naïve Bayes) on the holdout test set. The XGBoost Raw model showed the strongest performance among the comparison models (AUC = 0.848).

Both the CatBoost and XGBoost models demonstrated a strong ability to discriminate between admitted and discharged patients. The CatBoost model achieved an AUC of 0.870 on the holdout test set with a PR AUC of 0.770. At the optimized probability threshold, CatBoost attained a sensitivity of 79.0%, specificity of 78.0%, PPV of 63.0%, NPV of 88.0%, and an F1 score of 0.701 (Table 2). The XGBoost (raw) model achieved an AUC of 0.848 with a sensitivity of 69.1% and specificity of 82.2% at its optimized threshold. These models outperformed the developed simple logistic regression model (AUC 0.802). The Random Forest model also showed strong performance (AUC 0.839), though it required imputation of missing values. Naive Bayes showed the weakest performance (AUC 0.752). Figure 1A shows the ROC, PR AUC, and calibration curves for the champion CatBoost model. Figure 1B shows the corresponding curves for the five additional models. Table 2 presents the complete set of evaluation metrics for all six models on the holdout test set.

**Table 2:** Comparison of model performance on the holdout test set. The CatBoost model demonstrated the strongest overall performance. Each model was evaluated at its individually optimized probability threshold (tuned on training data to maximize F1). AUC: area under the receiver operating characteristic curve; PR AUC: area under the precision-recall curve; PPV: positive predictive value; NPV: negative predictive value

Model	AUC	PR AUC	Accuracy	Sensitivity	Specificity	PPV	NPV	F1	Brier Score
CatBoost	0.870	0.770	0.780	0.790	0.780	0.630	0.880	0.701	0.1462
XGBoost Raw	0.848	0.725	0.780	0.691	0.822	0.650	0.848	0.670	0.1474
XGBoost Imputed	0.837	0.712	0.774	0.665	0.826	0.647	0.838	0.656	0.1518
Random Forest	0.839	0.711	0.762	0.747	0.769	0.607	0.864	0.670	0.1502
Logistic Regression	0.802	0.660	0.725	0.729	0.724	0.558	0.859	0.632	0.1638
Naïve Bayes	0.752	0.550	0.652	0.799	0.582	0.477	0.858	0.598	0.2461

Post-hoc calibration

To assess whether post-hoc recalibration would improve the probabilistic outputs of the champion CatBoost model, we applied Platt scaling (sigmoid) fitted on a held-out 20% stratified calibration split of the training cohort (n = 124,146; 32.4% admitted). Calibrator parameters were A = 1.1019, B = −0.7398. On the 2024 holdout test set (n = 183,789), Platt scaling reduced the Brier score from 0.1463 to 0.1342 (an 8.2% relative improvement) and reduced log loss from 0.4446 to 0.4119. The calibration intercept improved from −0.692 (indicating systematic over-prediction of admission probability) to 0.018 (near-perfect), and the calibration slope improved from 1.057 to 0.960 (closer to the ideal value of 1.0). Isotonic regression produced essentially identical improvements (Brier 0.1342, slope 0.955, intercept 0.016). Platt scaling was preferred for any future clinical deployment because it requires only a two-parameter sigmoid transformation, is less prone to overfitting on smaller calibration sets, and preserves the monotonic ordering of risk scores. Discrimination metrics (AUC, sensitivity, specificity at the F1-optimized threshold) are unchanged by post-hoc calibration because the sigmoid transformation is monotonic; the value of recalibration is in producing reliable predicted probabilities for clinical interpretation. Appendix 2 reports the full set of pre- and post-calibration metrics. The corresponding reliability curves are shown in Appendix 3.

Feature importance and population-level explainability

**Figure 2:**
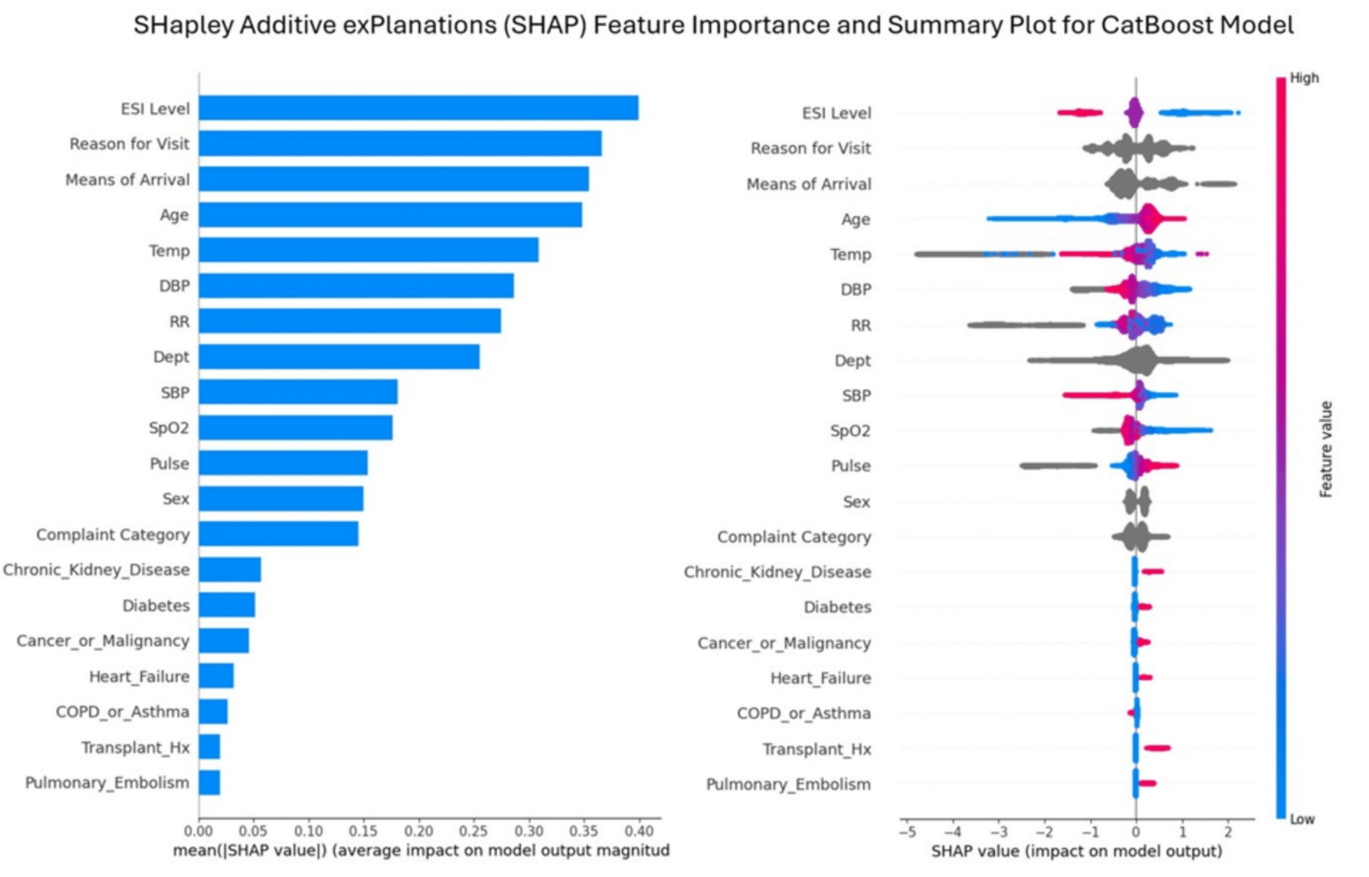
Top 20 predictor features ranked by SHAP-derived importance and SHAP tree explainer summary plots for the CatBoost model. Top 20 predictor features ranked by SHAP-derived importance in the CatBoost model, and SHAP tree explainer summary plots. Triage acuity (ESI level) was the most influential feature, followed by vital signs (heart rate, oxygen saturation, respiratory rate, blood pressure), complaint category, and mode of arrival. Comorbidities such as active cancer and heart failure had comparatively smaller influences. For the SHAP tree explainer summary plots, each point represents one prediction; feature values are color-coded, and horizontal position indicates the SHAP value (impact on model output). The Dept feature represents which of the three sites the patient visited. Features are ordered by overall importance. DBP: diastolic blood pressure, SBP: systolic blood pressure, RR: respiratory rate, Dept: ED site at which patient was seen

**Figure 3:**
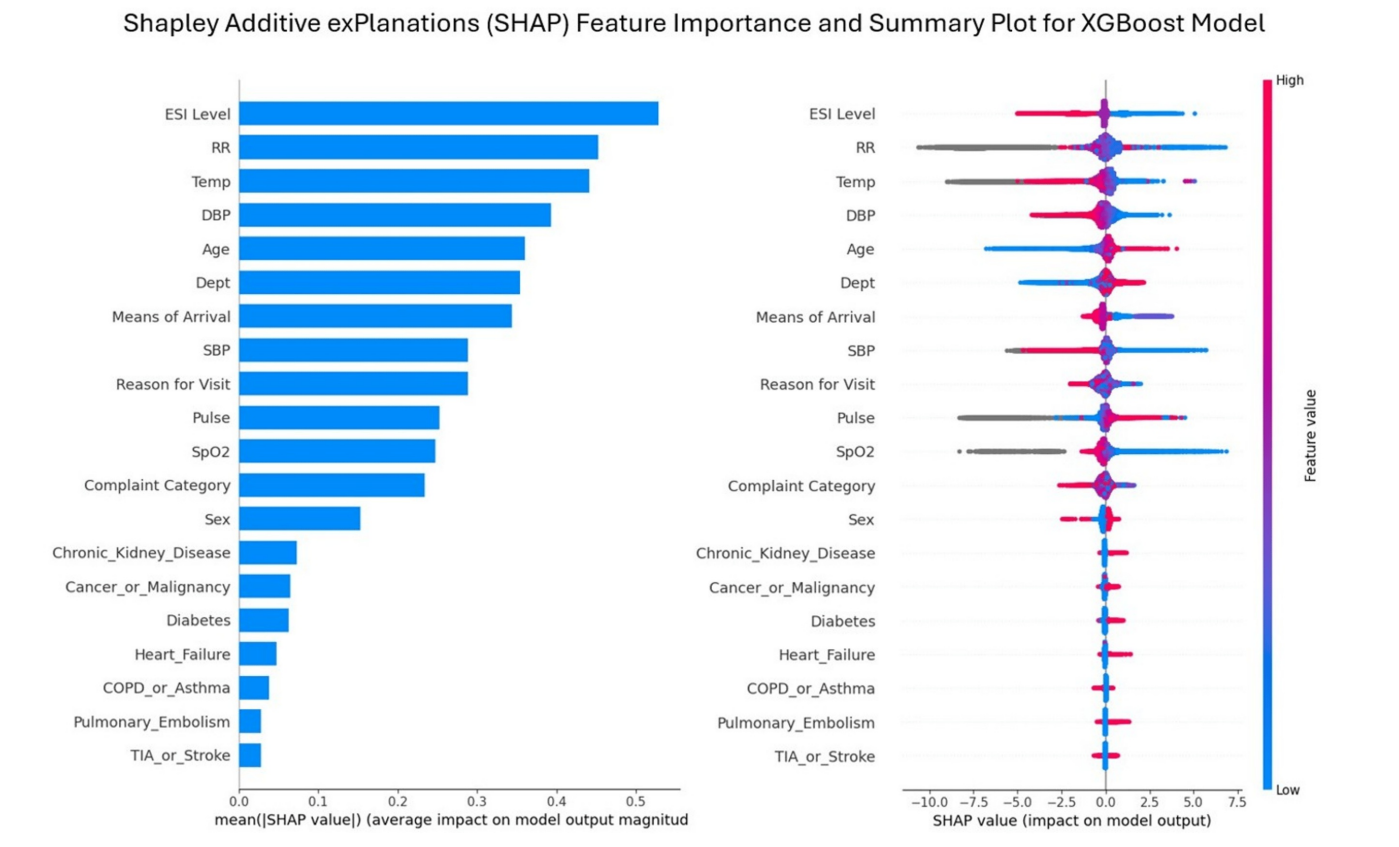
Top 20 predictor features ranked by SHAP-derived importance in the XGBoost Raw model, and SHAP tree explainer summary plots. The feature importance hierarchy for the XGBoost model was similar to the CatBoost model, with ESI level as the dominant predictor, followed by vital signs and arrival mode. For the SHAP tree explainer summary plots, each point represents one prediction; feature values are color-coded, and horizontal position indicates the SHAP value (impact on model output). The Dept feature represents which of the three sites the patient visited. Features are ordered by overall importance. DBP: diastolic blood pressure, SBP: systolic blood pressure, RR: respiratory rate, Dept: ED site at which patient was seen

To understand each model's decision-making, we examined feature importance rankings using SHAP values. Figure 2 displays the top 20 features by importance from the CatBoost model. Triage acuity (ESI level) was the most impactful predictor of admission risk; patients assigned ESI level 1 or 2 were far more likely to require admission than those triaged as ESI 3 or 4. Complaint category and mode of arrival also featured prominently among the top predictors. Several vital signs were highly influential, including heart rate, oxygen saturation, respiratory rate, and blood pressure. Age appeared as a moderate predictor; older age increased admission risk, but was not as strong as acute physiologic variables. The XGBoost model (Figure 3) showed a similar feature importance hierarchy.

Among comorbidities, a history of malignancy (active cancer) was among the higher-ranking factors, suggesting that patients with cancer who visit the ED may be more prone to requiring admission. Chronic heart failure and chronic lung disease had lower importance, and diabetes and prior stroke history fell outside the top 10. This indicates that while comorbidities contribute a predictive signal, the models primarily rely on the acute presentation, which aligns with emergency medicine clinical reasoning.

While the majority of SHAP value directions are clinically intuitive (e.g., increased age, decreased blood pressure, or decreased oxygen saturation leading to increased admission risk), it is important to note that some directional correlations are less immediately explainable. This further highlights the need for explainability techniques to be demonstrated alongside ML prediction tools so that model reasoning can be evaluated independently of aggregate performance metrics.

Individual-level explainability

**Figure 4:**
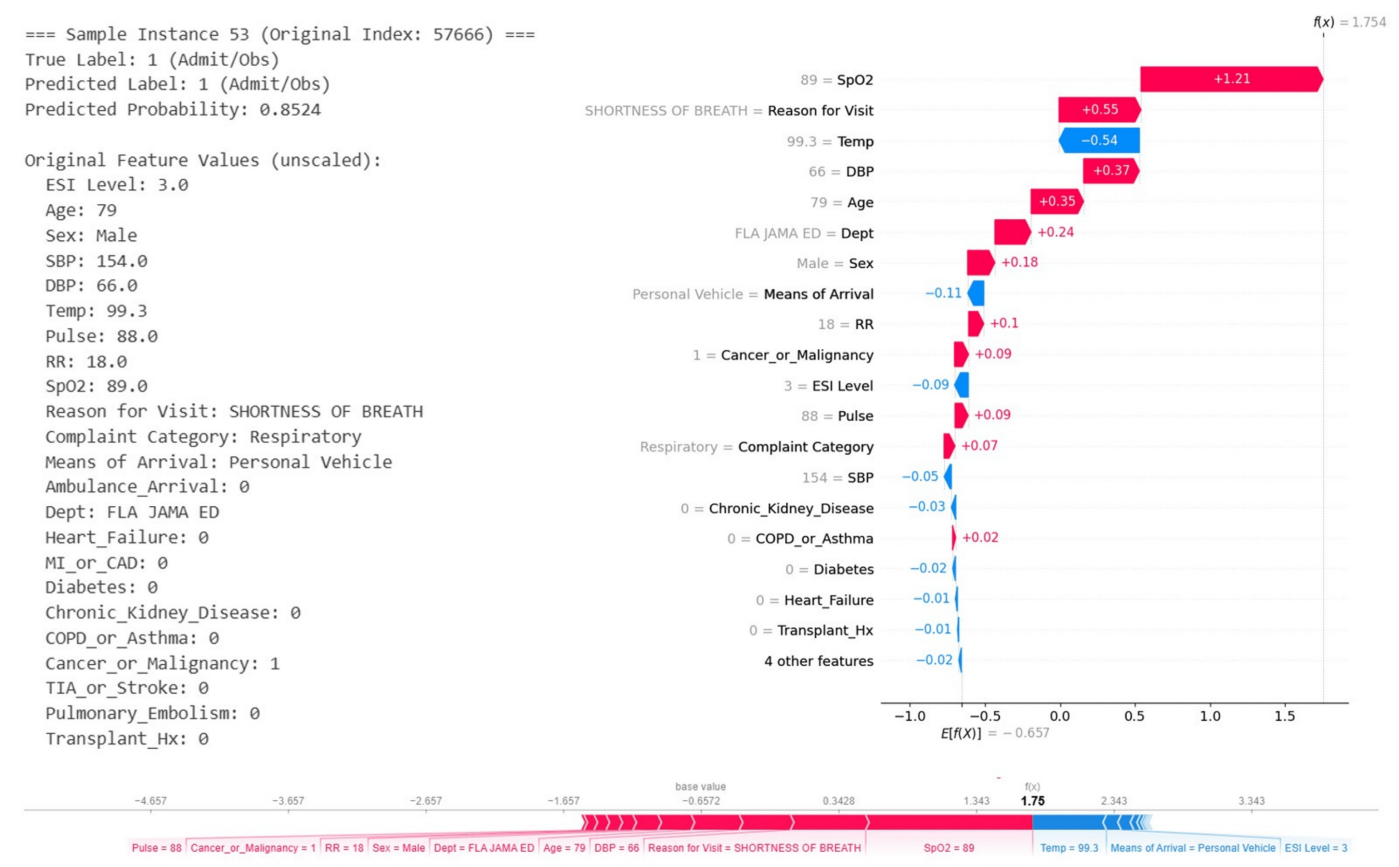
Individual prediction example 1 with SHAP waterfall and force plots for the CatBoost model. The f(x) threshold indicates the decision boundary. Red features push the prediction toward admission; blue features push toward discharge. The predicted probability was 0.8524 with the model threshold set to 0.5 for triggering a positive prediction alert. From data available at initial triage, the CatBoost model identified features of low O₂ saturation (89), patient age (79), presenting complaint of shortness of breath, and diastolic blood pressure (66) to be influential in predicting this patient’s eventual admission. DBP: diastolic blood pressure, SBP: systolic blood pressure, RR: respiratory rate, Dept: ED site at which patient was seen

**Figure 5:**
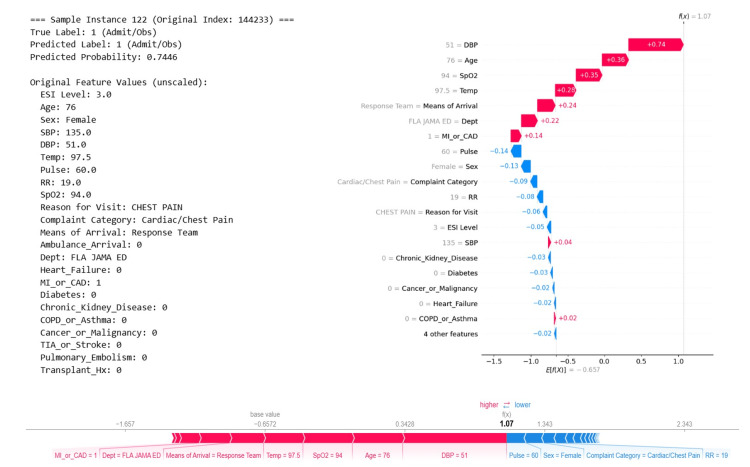
Individual prediction example 2 with SHAP waterfall and force plots for the CatBoost model. The f(x) threshold indicates the decision boundary. Red features push the prediction toward admission; blue features push toward discharge. The predicted probability was 0.7446 with the model threshold set to 0.5 for triggering a positive prediction alert. From data available at initial triage, the CatBoost model identified features of the patient's diastolic blood pressure (51), age (76), and O₂ saturation (94) to be the most influential in correctly predicting this patient's eventual admission. DBP: diastolic blood pressure, SBP: systolic blood pressure, RR: respiratory rate, Dept: ED site at which patient was seen

**Figure 6:**
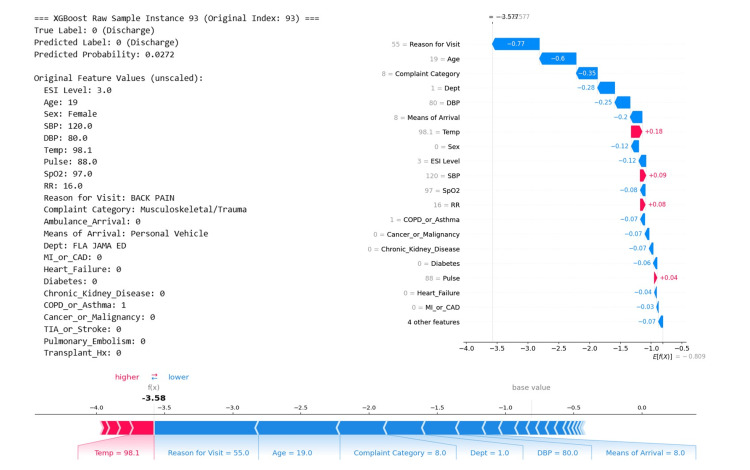
Individual prediction example 1 with SHAP waterfall and force plots for the XGBoost model. The f(x) threshold indicates the decision boundary. Red features push the prediction toward admission; blue features push toward discharge. The predicted probability was 0.0272 with the model threshold set to 0.5 for triggering a positive prediction alert. From data available at initial triage, the XGBoost model identified features of young age (19), back pain as the reason for visit, and lower acuity ESI (3) as influential features leading to a correct prediction of eventual patient discharge. DBP: diastolic blood pressure, SBP: systolic blood pressure, RR: respiratory rate, Dept: ED site at which patient was seen

**Figure 7:**
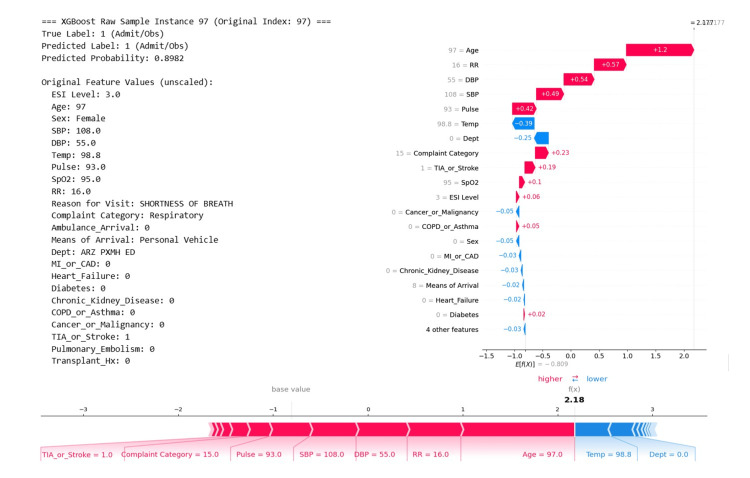
Individual prediction example 2 with SHAP waterfall and force plots for the XGBoost model. The f(x) threshold indicates the decision boundary. Red features push the prediction toward admission; blue features push toward discharge. The predicted probability was 0.8982 with the model threshold set to 0.5 for triggering a positive prediction alert. From data available at initial triage, the XGBoost model identified the patients' age (97), elevated respiratory rate (16), and diastolic (55) and systolic (108) blood pressures to be the most influential contributors towards a positive prediction. DBP: diastolic blood pressure, SBP: systolic blood pressure, RR: respiratory rate, Dept: ED site at which patient was seen

As an important focus of this study, we present individual-level explainability from the CatBoost and XGBoost models. Figures 4-7 show sample individual predictions with SHAP waterfall and force plots. The f(x) threshold indicates the decision boundary between admission and discharge classifications. It is important to recognize that SHAP values should not be interpreted as fixed effect sizes analogous to logistic regression coefficients; the impact of each variable on the prediction depends on the values of all other features for a given patient. All displayed predictions are from the holdout set, which is data unseen by the model during training. These plots demonstrate how individual-level explainability can serve as a "head-up display" for clinician review.

## Discussion

In this retrospective, multi-site study, we developed and validated ML models that predict hospital admission using data available at ED triage. The CatBoost and XGBoost models achieved good discriminative performance (AUC 0.870 and 0.848, respectively), substantially outperforming traditional logistic regression (AUC 0.802). Our findings demonstrate that triage factors, especially the nurse-assigned acuity level (ESI), vital signs, means of arrival, and complaint category, contain the essential information to accurately stratify patients' need for hospital admission. Importantly, we also demonstrated that individual-level SHAP explainability can serve as both a proxy for model reasoning and a type of "head-up display" for clinicians, providing transparent justification for each prediction. To our knowledge, this contribution of combined population-level and individual-level explainability applied to admission prediction from ED triage is relatively scarce in the current literature.

Comparison with prior literature

Our results corroborate and extend prior research on ED admission prediction. Parker et al. (2019) developed a model using eight variables and achieved an AUC of 0.825 [1]. Raita et al. (2019) used National Hospital Ambulatory Medical Care Survey (NHAMCS) data over five years and achieved an AUC of 0.82 for predicting admissions from triage data [13]. Fenn et al. (2021) developed multicenter prediction models and found that gradient-boosting trees demonstrated superior performance compared with logistic regression, achieving a validation AUC of 0.85 and an AUPRC of 0.692 for the admission task, similar to our gradient-boosted models [18]. Fenn et al. also performed prospective validation and delivered some explainability in the form of feature analysis. A study by Arnaud et al. is the only article to our knowledge to have recently demonstrated an individual-prediction-based method of explainability leveraging SHAP-based outputs and achieved an AUC of 0.83 using an artificial neural network [28]. Our study advances the literature by incorporating both population-level and individual-level explainability, enabling rigorous validation of model reasoning and facilitating direct comparison with clinician decision-making. Our results also confirm that gradient-boosting models perform strongest among the tested model types for admission prediction and that prediction of admission to inpatient-level care is achievable with good discrimination using structured triage data.

Clinical implications

Early recognition of likely admissions with ML has the potential to expedite bed requests, reduce ED boarding times, and improve overall throughput [29,30]. However, engagement with decision support tools has been historically slow and inefficient [22,24,25]. Individual-level explainability may play a critical role in promoting physician utilization of AI-based decision support tools [22,31,32]. A recent systematic review examining trust in artificial intelligence-based clinical decision support systems among health care workers found that lack of transparency and perceived "black-box" reasoning were among the most cited barriers to clinician trust and adoption [22]. Explainability and clear communication of how predictions are generated were consistently associated with higher reported trust and greater intention to use AI tools in practice. Our principal contribution is the demonstration of these explainability techniques at both the population level (SHAP summary plots) and the individual level (SHAP waterfall and force plots). These methods could be presented as a "heads-up display" for practitioners to review alongside model predictions as a conceptual framing for how individual SHAP explanations could be integrated into the EHR; the operational utility of this framing has not yet been empirically evaluated. Models similar to those developed here have already entered prospective validation at other institutions; thus, we place an emphasis on demonstrating explainability early in the development phase [16,22]. Moreover, the FDA Quality Management System Regulation guidelines were recently amended to reinforce transparency and interpretability as part of the ML models' validation process to ensure safe and effective use before deployment to clinical environments [27]. This shift emphasizes the need to incorporate explainability interfaces early into model evaluation from both a clinician trust and a regulatory compliance perspective. Our demonstrated methods present a promising framework for researchers to plan for adoptability and compliance in the development of their models. By presenting patient-specific SHAP explanations alongside each admission prediction, our model offers physicians real-time insight into the key drivers of risk for a specific patient. As such, individual-level explainability is hypothesized to function as an operable technical feature and a candidate trust-building mechanism, pending prospective evaluation with clinicians [33-35].

As demonstrated in our individual explanations through SHAP waterfall and force plots, some model predictions aligned well with clinical reasoning; however, other predictions were harder to explain, complicating the use of such models as a decision support tool. For this reason, a method of examining the influence of input features for each prediction is paramount. Providing clinicians with a more complete understanding of why a model makes an individual prediction may lead to better-informed use of ML-based decision support tools, improved allocation of resources, and better patient care.

From an implementation standpoint, such models may be deployed within the EHR to generate a risk score for each new ED arrival. Many modern EHR systems can host custom ML models and display risk scores along with individual explainability of a generated score on the ED track board or a triage dashboard. The aggregate predicted admission rate at any point in time may also be valuable to capacity-management staff and bed-control workflows, complementing per-patient triage decisions made by clinicians. Anticipated demand on inpatient beds is itself a useful product of admission-prediction models and may be the most readily adoptable use case in operational settings.

SHAP-based explanations have known limitations that warrant clinical caution. SHAP values reflect each feature's contribution to a model's prediction, not causal relationships between that feature and the underlying outcome. Explanations can also be unstable in the presence of strongly correlated input features, where the attributed contribution may shift between two correlated variables across different patients or different model retrains. Finally, SHAP outputs can be over-interpreted by clinicians as definitive justifications for a prediction; in practice, individual SHAP plots should be paired with appropriate uncertainty communication, clinician training on the nature of SHAP outputs, and recognition that the underlying model is a probabilistic tool. These limitations do not invalidate the use of SHAP for transparent model evaluation but warrant explicit acknowledgement and clinician-facing communication in any future deployment.

We recognize potential downsides of clinical deployment that warrant explicit consideration. First, over-reliance on a predictive model can reduce independent clinical reasoning and erode skills if the model is consulted reflexively. Second, alert fatigue is a well-documented failure mode of EHR-embedded decision support; introducing a continuous admission risk score on the triage board may add to existing alert burden unless the display is carefully integrated. Third, when the model and the bedside clinician disagree, there is a risk of inappropriate bias pressure in either direction, either anchoring the clinician to a wrong model prediction or undermining a correct clinical judgment. Mitigation strategies include user-centered design, optional rather than mandatory display, periodic review of model-clinician disagreement cases, and ongoing model monitoring for drift.

Limitations

This study has several limitations. Although our cohort spans three geographically distinct academic medical center campuses, all three EDs operate under a shared enterprise EHR, common admission protocols, common nurse-training curricula for triage acuity assignment, and an overlapping patient case mix. Performance characteristics observed in this study should not be assumed to transfer to institutions outside our health system, particularly those with different workflows, EHR configurations, or population case mix. Prospective external validation at an unrelated institution is a necessary next step before broader clinical deployment.

Second, we used only structured triage data. Inclusion of unstructured data, such as triage nurse notes or chief complaint free text, could improve prediction, as the chief complaint carries acuity information not fully captured by ESI [15]. Our focus was to critically examine model performance with readily available structured data and to add explainability. Third, although we tuned the threshold to maximize F1, the optimal threshold may differ by application; some EDs may prefer higher sensitivity and tolerate more false alerts, while others may prioritize specificity.

We performed post-hoc Platt scaling as a sensitivity analysis (Appendices 2, 3). Calibration substantially improved with Platt scaling, while discrimination was preserved by construction (monotonic transformation). If this model were deployed clinically, the Platt-scaled probabilities should be used for any application that interprets the predicted probability as a clinical risk.

Although all input features were captured at or before triage, structured categorical fields such as complaint category and means of arrival may indirectly encode triage-nurse acuity judgment or site-specific routing patterns. Predictive performance attributable to these features should therefore be interpreted as performance of a model that incorporates triage clinical judgment, not performance of a feature set entirely independent of clinical reasoning.

We did not perform usability testing, clinician trust evaluation, or any prospective implementation study. All claims regarding clinician adoption, trust, and operational benefit are hypothesized and require future empirical evaluation.

Finally, our analysis of feature importance and SHAP values provides transparency but does not fully eliminate the "black box" concern of ML. However, we suspect that real-time clinical deployment will benefit from displaying individual-level explanations alongside predictions as modeled in this study to support clinician trust.

Future directions

As this study is retrospective in nature, prospective validation is needed to assess both the performance and benefit of the demonstrated explainability methods. While prior work has demonstrated both the benefits and challenges of integrating predictive tools into workflow, the coupling of well-performing ML models with explainable outputs may improve clinician comprehension and trust, thereby promoting increased engagement and more meaningful evaluation during prospective implementation.

A formal assessment of clinician trust in ML decision support systems that incorporate individual explainability techniques is necessary to reveal if adding explainability displays is truly beneficial. Following prospective validation and data collection on physician-perceived utility would be the integration of these explainability techniques into EHR workflows. Collaboration with clinical informatics and development of a focused, user-centered design that incorporates individual explainability along with a continuous risk score may increase utility and engagement with such a model, with the goal of reducing cognitive load for clinicians [36,37].

Other potential directions include incorporating chief complaint text via natural language processing, integrating early laboratory results, and studying generalizability across external datasets. Finally, this study might be expanded to include time series data following initial triage data of patients beyond arrival to see if this might improve performance by capturing evolving clinical statuses.

## Conclusions

We developed and evaluated six ML models that leverage triage data to predict hospital admission from the ED. The champion CatBoost model demonstrated an AUC of 0.870 with balanced sensitivity (79.0%) and specificity (78.0%) at the optimized threshold. Triage acuity (ESI level), vital signs, complaint category, and means of arrival were the dominant predictors, underscoring the continued importance of fundamental clinical measurements in risk stratification. Population-level and individual-level SHAP explainability techniques provide a transparent framework for evaluating model reasoning that may support clinician trust and facilitate integration into ED practice. As ED volumes and operational pressures continue to rise, data-driven tools augmented with explainability can support clinicians in delivering the right level of care at the right time. Future prospective studies and physician surveys utilizing these explainability techniques will be required to determine whether interpretable model outputs translate into measurable changes in clinician decision-making, workflow efficiency, or patient outcomes.

## Figures and Tables

**Figure 8:**
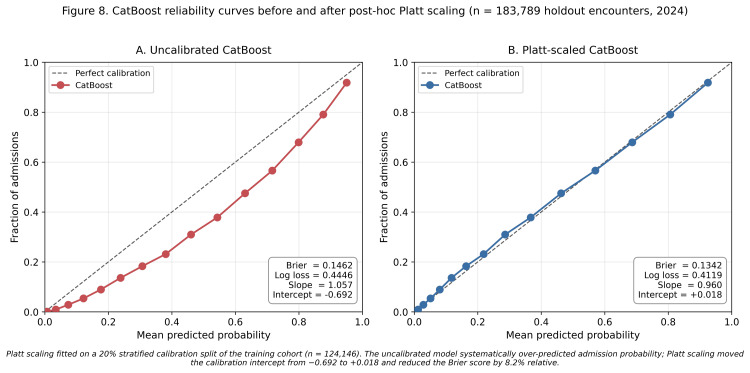
CatBoost reliability curves before and after post-hoc Platt scaling. Reliability curves for the champion CatBoost admission-prediction model before (Panel A) and after (Panel B) post-hoc Platt scaling on the 2024 holdout test set (n = 183,789). Platt scaling, fitted on a 20% stratified calibration split of the training cohort, moved the calibration intercept from −0.692 to +0.018 and reduced the Brier score from 0.1463 to 0.1342 (8.2% relative improvement). Discrimination metrics are unchanged by the monotonic sigmoid transformation. Full pre- and post-calibration metrics are reported in Appendix 2.

## Appendices

Appendix 1

**Table 3:** Final tuned hyperparameters for all six models. All models were trained on the same 80/20 stratified train/validation split of the 2020-2023 training cohort (random_state=42). Final evaluation on the temporally distinct 2024 holdout. Categorical preprocessing differs per model: CatBoost handles categoricals natively; all comparator models use label-encoded integer codes. For Logistic Regression, Random Forest, Naive Bayes, and XGBoost-Imputed, numeric features were median-imputed (sklearn SimpleImputer) and then StandardScaler-transformed.

Model	Hyperparameters	Random seed
CatBoost (champion)	iterations=800; depth=6 (default); learning_rate=0.03; l2_leaf_reg=3 (default); loss_function='Logloss'; eval_metric='Logloss'; auto_class_weights='Balanced'; verbose=0	42 (train/val split); CatBoost defaults for internal randomization
XGBoost (Raw)	n_estimators=800; max_depth=6 (default); learning_rate=0.3 (default); use_label_encoder=False; eval_metric='logloss'; missing=NaN (native handling)	42
XGBoost (Imputed)	Same as XGBoost (Raw); applied to median-imputed and StandardScaler-transformed numeric features	42
Random Forest	n_estimators=100 (default); max_depth=None (default); class_weight='balanced'	42
Logistic Regression	penalty='l2' (default); C=1.0 (default); solver='lbfgs' (default); max_iter=1000	42
Naive Bayes (GaussianNB)	var_smoothing=1e-9 (default)	n/a

Appendix 2

**Table 4:** Pre- and post-calibration metrics for the champion CatBoost model (n = 183,789 holdout). Platt scaling and isotonic regression were each fitted on a 20% stratified calibration split of the training cohort (n = 124,146; 32.4% admitted; random seed 42) and applied to the temporally distinct 2024 holdout test set. Platt scaling parameters: A = 1.1019, B = −0.7398, where P_calibrated = 1 / (1 + exp(−(A · logit(p) + B))). The uncalibrated model systematically over-predicted admission probability (calibration intercept −0.692); post-hoc Platt scaling moved the intercept to +0.018 (near-perfect) and reduced the Brier score by 8.2% relative. Isotonic regression produced essentially identical improvements. Discrimination metrics (AUC, sensitivity, specificity, PPV, NPV, F1) are unchanged by post-hoc calibration because the sigmoid transformation is monotonic.

Method	Brier	Log loss	Cal. slope	Cal. intercept	Δ Brier vs uncalibrated
Uncalibrated (champion CatBoost)	0.1463	0.4446	1.057	−0.692	(reference)
Platt scaling (sigmoid)	0.1342	0.4119	0.960	+0.018	−0.0120
Isotonic regression	0.1342	0.4120	0.955	+0.016	−0.0120

Appendix 3
